# Examining the Level of Knowledge of Teachers About Asthma, Diabetes and Epilepsy in Children: A Systematic Review

**DOI:** 10.3390/children13010091

**Published:** 2026-01-08

**Authors:** Aleksandar Petrušić, Miloš N. Milosavljević, Mladen Pavlović, Miroslav M. Sovrlić, Milos Stepovic, Nevena Folic, Valentina Marinković, Andrijana Milošević Georgiev

**Affiliations:** 1 Department of Social Pharmacy and Pharmaceutical Legislation, Faculty of Pharmacy, University of Belgrade, 11158 Belgrade, Serbia; hadzipetrusic@icloud.com (A.P.); valentina.marinkovic@pharmacy.bg.ac.rs (V.M.); andrijana.milosevic@pharmacy.bg.ac.rs (A.M.G.); 2 Department of Pharmacology and Toxicology, Faculty of Medical Sciences, University of Kragujevac, 34000 Kragujevac, Serbia; milosavljevicmilos91@gmail.com; 3 Department of Surgery, Faculty of Medical Sciences, University of Kragujevac, 34000 Kragujevac, Serbia; drmpavlovic@gmx.com; 4 Department of Pharmacy, Faculty of Medical Sciences, University of Kragujevac, 34000 Kragujevac, Serbia; sofke-ph@hotmail.com; 5 Department of Anatomy, Faculty of Medical Sciences, University of Kragujevac, 34000 Kragujevac, Serbia; 6 Department of Pediatrics, Faculty of Medical Sciences, University of Kragujevac, 34000 Kragujevac, Serbia; nevena.folic@yahoo.com; 7 Pediatric Clinic, University Clinical Center Kragujevac, 34000 Kragujevac, Serbia

**Keywords:** teachers, children, chronic disease, asthma, diabetes mellitus, epilepsy

## Abstract

**Background/Objectives**: Asthma, type 1 diabetes mellitus (T1DM), and epilepsy are prevalent chronic diseases among school-aged children, affecting safety, attendance, and academic performance. This systematic review evaluated school teachers’ knowledge, attitudes, and preparedness regarding these conditions and identified gaps that hinder effective management and inclusion. **Methods**: Following PRISMA guidelines, PubMed, Cochrane Library, Scopus, and Google Scholar were searched between 20 September and 9 October 2025. Forty-nine quantitative cross-sectional studies assessing teachers’ knowledge, attitudes, or preparedness toward asthma, T1DM, or epilepsy were included. The AXIS tool assessed methodological quality, focusing on clarity of objectives, sample justification, ethical transparency, and instrument validation. **Results**: Teachers’ knowledge was generally moderate and varied by region. Studies on epilepsy (n = 21) highlighted misconceptions and limited understanding of seizure first aid. Diabetes studies (n = 9) indicated moderate awareness but insufficient preparedness for hypoglycemia and insulin management. Asthma studies (n = 19) revealed inconsistent knowledge, particularly regarding symptom recognition and emergency response. AXIS assessment identified recurring limitations, including unjustified sample sizes, limited instrument validation, and poor reporting of non-responders. **Conclusions**: These findings emphasize the need to enhance school preparedness through targeted, evidence-based teacher training, clear health policies and emergency protocols, awareness and inclusion initiatives, improved collaboration among teachers, parents, and healthcare providers, and strengthened school health infrastructure. Addressing these areas is critical to ensure safe, inclusive, and supportive learning environments for children with chronic illnesses.

## 1. Introduction

Asthma, type 1 diabetes mellitus (T1DM), and epilepsy are three of the most common chronic diseases among school-aged children. Asthma is one of the most common chronic diseases among school-aged children, affecting approximately 8.3% of primary and secondary school students in the United States [1]. The prevalence of T1DM in Europe among children and adolescents under 20 years of age is approximately 419,000, with an annual incidence of 31,000 new cases, making Europe the region with the highest number of affected children and the highest annual incidence of T1DM worldwide [2]. Additionally, the estimated prevalence of epilepsy among children and adolescents in Europe is 0.9 million, with a prevalence rate of 4.5 to 5.0 per 1000, indicating a significant public health concern [3].

Asthma represents a leading cause of school absenteeism, with an estimated 36,000 children worldwide missing school every day due to asthma-related problems [4,5]. On average, children with asthma miss 2.3 more school days per year compared to their healthy peers and demonstrate poorer academic achievement [6,7,8]. Children with T1DM face several challenges in the school environment, including limited access to insulin and a high proportion of teachers who are not trained in diabetes management [9]. These challenges may lead to interruptions in glycemic control, increased risk of acute complications such as hypoglycemia, and reduced participation in school activities [10]. Similarly, children with epilepsy often experience social stigma, anxiety, and difficulties with academic engagement and peer relationships due to teachers’ uncertainty regarding seizure management and emergency response [11]. Those groups of children may have additional limitations in extracurricular activities, field trips, and sports participation, highlighting the need for comprehensive support within schools [12].

Considering that children spend about 30% of their day in school, educational institutions play a crucial role in ensuring proper care and support for students with asthma, T1DM, and epilepsy [1,13]. In developed countries such as the United States, one of the major advances in supporting children with chronic illnesses has been the employment of school nurses, who play a pivotal role in promoting child health, training teachers, and ensuring the safety of children during school hours [14,15,16]. However, only 40% of U.S. schools employ full-time nurses, while 25% have none [17]. In schools without medical staff, the responsibility for recognizing and responding to health issues often falls on teachers, who may lack adequate training and confidence to manage these conditions effectively [18]. These challenges underscore the need to assess teachers’ knowledge and preparedness to support students with chronic illnesses. These data highlight the necessity for comprehensive educational programs that cover asthma, T1DM, and epilepsy to ensure safe, inclusive, and supportive school environments for all students.

While acknowledging that multiple chronic health conditions can affect school functioning in children, asthma, type 1 diabetes mellitus, and epilepsy were selected for this review because of their high prevalence in school-aged populations, substantial healthcare and societal costs, and shared clinical and educational challenges. These conditions impose a considerable public health burden through ongoing medical care, frequent healthcare utilization, school absenteeism, and indirect costs related to reduced academic participation and parental work loss [5,6]. In addition, all three conditions are associated with acute and potentially life-threatening events that may occur during school hours and require prompt recognition and intervention [9,11,14]. In many school settings, particularly those without full-time medical personnel, teachers are often responsible for the initial response, making adequate knowledge, preparedness, and confidence essential for ensuring student safety, inclusion, and continuity of education [18].

This systematic review aims to evaluate, based on the available literature, the level of knowledge, attitudes, and preparedness of school staff regarding the symptoms, management, prevention, and treatment of asthma attacks, T1DM, and epilepsy in school-aged children, with particular emphasis on comparing these competencies across the three chronic conditions. Specifically, the review will focus on:

1. Assessing teachers’ understanding of the symptoms, triggers, and management procedures for children with asthma, T1DM, and epilepsy.
2. Identifying gaps in knowledge and confidence related to managing acute episodes during school hours.
3. Examining the effectiveness of existing educational interventions designed to improve teachers’ competencies in managing these chronic conditions.

By addressing these objectives, the review seeks to inform the development of targeted educational programs and policy measures aimed at improving care, safety, and inclusion for children with asthma, T1DM, and epilepsy within the educational environment.

## 2. Materials and Methods

This systematic review adhered to the Preferred Reporting Items for Systematic Reviews and Meta-Analyses (PRISMA) 2020 statement (PRISMA Group, Oxford, UK) [19]. The study protocol was registered in the International Prospective Register of Systematic Reviews (PROSPERO; Centre for Reviews and Dissemination, University of York, York, UK; registration ID: CRD420251169339).

### 2.1. Eligibility Criteria

The systematic review included original research studies conducted among primary and secondary school teachers that examined their knowledge, attitudes, or preparedness regarding children with bronchial asthma, type 1 diabetes mellitus (T1DM), or epilepsy. Eligible studies were those that assessed teachers’ knowledge before the implementation of any educational or training intervention related to the management of these chronic conditions. Only studies that applied quantitative research methods, published in English, were included in the review.

Studies were excluded if they were narrative or systematic reviews, meta-analyses, or if they focused exclusively on populations other than teachers, such as students, parents, or school nurses. studies that evaluated teachers’ knowledge after participation in educational or intervention programs, as well as studies presenting qualitative methodologies, were also excluded. Additionally, papers published in languages other than English were omitted from the final analysis.

### 2.2. Information Source

The literature search was conducted between 20 September and 9 October 2025 using PubMed (MEDLINE), the Cochrane Library, Scopus, and Google Scholar, with no restrictions on publication date.

### 2.3. Search Strategy

For the generation of keywords, the official MEDLINE/PubMed thesaurus Medical Subject Headings (MeSH) was used [20]. Initially, the following keywords directly related to the study objective were entered into the MeSH database, using a combination of different keywords with a Boolean operator: school teacher, knowledge, and asthma/diabetes/epilepsy. By combining these terms, the following search strategy was developed and applied to the selected biomedical databases: (school teacher OR elementary school teacher OR middle school teacher OR high school teacher) AND (knowledge OR awareness) AND (asthma OR diabetes OR epilepsy).

### 2.4. Selection Process

**Figure 1:**
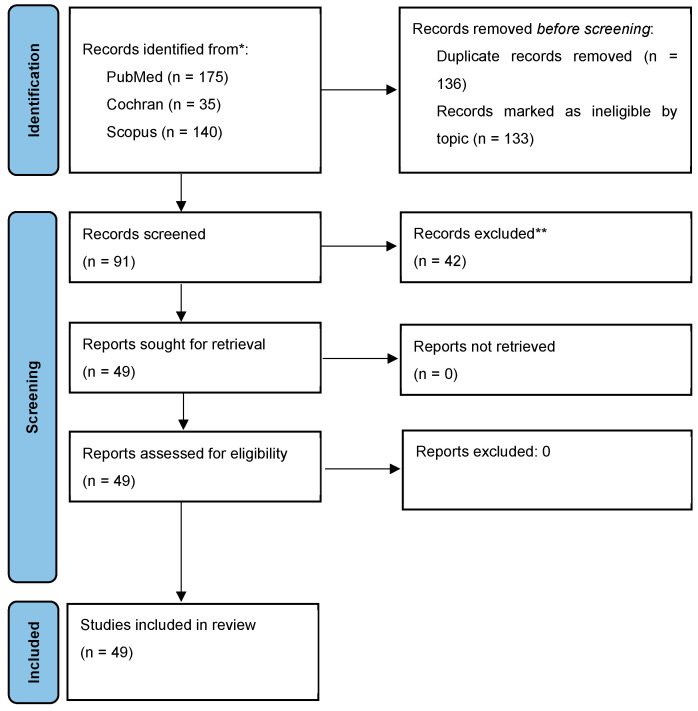
PRISMA Flow chart; * databases from which the studies were identified; ** records excluded after title and abstract screening due to irrelevance to the study population, study design, or outcomes.

The number of articles found in each database, like the process of removing duplicates and the selection of articles, is presented in the PRISMA flow diagram (Figure 1) [21]. Records excluded after title and abstract screening (n = 42) were removed due to irrelevance to the study population (non-teachers), assessment of post-intervention outcomes, qualitative study design, or outcomes not related to teachers’ knowledge of asthma, diabetes, or epilepsy. The total number of articles included in this systematic review was 49.

### 2.5. Data Collection Process

Two reviewers (MS and AP) independently screened each record by title and abstract, retrieved each report, and subsequently removed duplicate studies for further analysis. Where necessary, a third reviewer (MM) was contacted to help resolve disagreements between screeners.

### 2.6. Data Items

From the studies included in the final analysis, the following variables were extracted: the type and country of the study, the number and type of schools involved (primary or secondary), the number of participating teachers, their demographic characteristics (gender and age), the instrument used to assess teachers’ knowledge regarding bronchial asthma, type 1 diabetes mellitus (T1DM), and epilepsy in children, and the overall evaluation or scoring of teachers’ knowledge related to the management of these chronic conditions in the school setting.

### 2.7. Risk of Bias Assessment

The AXIS tool (Appraisal tool for Cross-Sectional Studies) is a standardized instrument developed to assess the quality and risk of bias in cross-sectional studies. It was designed by Downes et al. (2016) to provide a comprehensive and structured framework for evaluating the methodological soundness of observational research [22]. The tool consists of 20 items, divided into several key domains, and figures presenting the AXIS assessment for each disease were provided in the Supplementary Materials.

Each item in the AXIS checklist is scored as “Yes,” “No,” or “Don’t know,” allowing reviewers to systematically identify methodological strengths and weaknesses. Unlike some other critical appraisal tools, AXIS does not produce a numerical summary score but rather encourages qualitative assessment and justification of each criterion.

The AXIS tool is particularly suitable for systematic reviews conducted under the PRISMA framework, especially when the included studies are predominantly cross-sectional in design, a common feature in research exploring knowledge, attitudes, and practices among health professionals or educators. Its structured approach ensures transparency and reproducibility in the appraisal process, which are core principles of the PRISMA methodology. The results of the assessment for each topic of interest are presented in the results with emphasizing the strengths and weaknesses of the research design.

### 2.8. Synthesis Method

This systematic review employs qualitative synthesis and quantitative summary to present the main findings of the included studies, organized according to the three conditions of interest: asthma, type 1 diabetes, and epilepsy. Teachers’ attitudes are summarized using mean percentage values derived from self-reported assessments.

## 3. Results

### 3.1. AXIS Assessment

The AXIS assessments of studies on epilepsy, diabetes, and asthma consistently indicate that the included research generally had clearly defined objectives and appropriate study designs, providing a solid foundation for investigating knowledge, attitudes, and practices among teachers and students. Across all three disease areas, reporting of methods, basic data, and analytical procedures was largely adequate, and ethical approval was commonly documented, supporting the internal validity of the findings. However, recurring methodological limitations were identified across studies, particularly in domains related to sampling and response handling. Asthma studies demonstrated overall appropriate designs and coherent reporting, but frequently lacked sample size justification, comprehensive non-responder analysis, and consistent validation of measurement instruments, with several studies providing limited information on response rates or the representativeness of their samples (Figure S1). Diabetes studies showed comparatively stronger methodological performance, with robust reporting of outcomes, statistical analyses, and ethical transparency; nevertheless, concerns persisted regarding sample representativeness and insufficient reporting on non-responders, which may affect the generalizability of results despite generally adequate measurement validity (Figure S2). In epilepsy studies, although aims, study designs, and reporting clarity were consistently strong, sample size justification was often absent, non-response handling was minimal, and instrument validation was inconsistently reported, increasing the risk of selection bias and limiting confidence in population-level inferences (Figure S3). Collectively, these AXIS evaluations suggest that while the included studies are of moderate methodological quality and provide valuable insights, there is a consistent need for clearer sample size justification, improved representativeness of study populations, systematic handling and reporting of non-response, and the use of validated measurement instruments to enhance the reliability, comparability, and applicability of future research findings.

### 3.2. Findings from Studies on Asthma

A total of 19 studies assessing school teachers’ knowledge and preparedness regarding asthma were included [23,24,25,26,27,28,29,30,31,32,33,34,35,36,37,38,39,40,41], spanning multiple countries: the United States, Brazil, Iraq, Jordan, Nigeria, Iran, Turkey, Egypt, Saudi Arabia, South Africa, Spain, Malta, and Norway. Most studies employed cross-sectional survey designs, with one mixed-method sequential explanatory study in Brazil [24]. Sample sizes ranged widely, from 65 teachers in Bronx elementary schools [26] to 4679 teachers across pre-school, primary, and secondary schools in Spain [40]. The number of schools included varied considerably, with some studies not reporting the exact number, and others included 4–208 schools.

Teachers were predominantly female across the studies, with proportions ranging from 58.5% to 95%, and the mean age ranged from 38 to 44.6 years, where reported. Elementary schools were the most common study setting, although some studies included secondary and high school teachers [23,28,35,39,40].

All studies utilized self-administered questionnaires to assess asthma knowledge or preparedness, though instruments varied. Some studies used validated questionnaires, such as the Newcastle Asthma Knowledge Questionnaire [24,36,40] or adaptations based on NHLBI (National Heart, Lung, and Blood Institute) guidelines [26,41] or KASE-AQ (Knowledge of Asthma Self-Efficacy Questionnaire) [30]. Other studies employed custom-designed questionnaires with reliability coefficients reported [29,31], and some including a 55-item face-to-face questionnaire administered via a mobile application [32], partially validated Likert-scale instruments [33], and electronic self-reported surveys [37].

Knowledge outcomes revealed considerable variation across regions. In the United States, 60.6% of teachers reported not feeling well prepared to manage asthma [23], and 68% felt uncomfortable assessing or managing asthma attacks in Bronx elementary schools [26]. In South Africa, 38.5% of teachers scored below 50%, indicating limited asthma knowledge [32]. In Spain, mean knowledge scores corresponded to approximately 52% correct answers, with particularly low recognition of symptoms, triggers, and rescue medications [34], while only 44.5% of teachers reported knowing how to manage an asthma attack and 54% did not know how to administer asthma medication [35]. In Brazil, 63.8% of teachers had unsatisfactory knowledge scores [24], and misconceptions regarding asthma triggers were common, with only 4.2% correctly identifying the three main symptoms [36], whereas in Iraq, teachers had a mean total confidence score of 72.44% in managing children with asthma [25]. Jordanian teachers answered nearly 50% of asthma first-aid questions incorrectly [27]. Nigerian teachers demonstrated poor knowledge in 48.1% of participants [28]. In Iraq, teachers reported relatively high confidence scores (72.44 ± 13.61) and moderate knowledge levels [38]. In Iran, teachers’ mean knowledge score was 12 out of 16 (intermediate level) [29], while Turkish teachers achieved approximately 74.3% of the maximum knowledge score [30] and in other study mean score of approximately 60.4% [33], while teachers in Malta demonstrated low overall knowledge, with a mean score of 5.5 ± 3.3 out of 14 points [37]. Finally, in Egypt and Saudi Arabia, 44.8% of teachers demonstrated unsatisfactory knowledge [31] while 59.6% of teachers demonstrated high overall asthma awareness, particularly regarding symptoms and treatment [40]. In Norway, only 25.9% of teachers reported sufficient knowledge to teach pupils with asthma, while 89.4% expressed a need for additional training [39] (Table 1).

### 3.3. Findings from Studies on Diabetes

A total of 9 studies assessing school teachers’ knowledge and attitudes regarding diabetes were included [42,43,44,45,46,47,48,49,50], conducted across Saudi Arabia, Portugal, Turkey, Poland, Germany, Spain, and the United Kingdom. Most studies employed cross-sectional survey designs [42,44,45,46,47], including large-scale descriptive observational and online cross-sectional surveys [48,49,50], while one used an experimental pre-test/post-test design in Portugal [43]. Sample sizes ranged from 129 teachers in the Portuguese study [43] to 1054 teachers in Turkey [44] with substantially larger samples reported in Spain (765 teachers) and Turkey (42,349 teachers) [48,49]. The number of schools included varied, with some studies not reporting exact numbers, while others included 12–18 schools, 44 public schools in Spain [48], and 40 primary and secondary schools in the United Kingdom [50].

Teachers were predominantly female across all studies, with proportions ranging from 56% to 100%, and mean ages ranged from 37.9 to 45–54 years, where reported. Study settings typically included both elementary and high schools, with one study focusing exclusively on female schools in Saudi Arabia and Spain [46,48]. All studies utilized self-administered questionnaires to assess diabetes knowledge and attitudes. A large 55-item online questionnaire with acceptable reliability (α = 0.71) was applied in Turkey [49], while earlier studies relied on shorter self-administered questionnaires combining knowledge and attitude domains [50]. Several instruments were validated, including a self-completed questionnaire with high reliability (α = 0.926) in Portugal [43] and the 20-item Test of Diabetes Knowledge for Teachers in Saudi Arabia [46]. Other studies used author-developed tools, such as a 29-item knowledge test in Poland [45].

Knowledge outcomes were generally moderate across studies. In Saudi Arabia, teachers achieved an average knowledge score of 4.4 out of 7 (moderate) and a favorable attitude score of 3.5 out of 5 [42]. Portuguese teachers’ pre-test scores were moderate at 10.8 out of 17 [43]. In Turkey, 47.6% of teachers had moderate knowledge, while 32.4% demonstrated low knowledge [44]. Polish teachers’ median score was 69% [45], and Saudi female teachers scored 66% on the diabetes knowledge test [46]. In Spain, 58% of teachers demonstrated insufficient knowledge, 36.9% had basic knowledge, and only 5.1% achieved a level compatible with effective student support [48]. In a large Turkish online survey, the mean knowledge score was 17.7 out of 39, indicating overall limited knowledge despite high awareness of diabetes prevention strategies [49]. In the United Kingdom, only 25% of teachers demonstrated adequate diabetes knowledge [50]. The German study did not explicitly report knowledge scores [47] (Table 2).

### 3.4. Findings from Studies on Epilepsy

A total of 21 studies assessing school teachers’ knowledge and attitudes regarding epilepsy were included [51,52,53,54,55,56,57,58,59,60,61,62,63,64,65,66,67,68,69,70], conducted across Saudi Arabia, Sudan, Iran, Niger, Gabon, Kuwait, Italy, Ethiopia, Brazil, Nigeria, Montenegro, Greece, as well as Nepal [69]. Most studies employed cross-sectional survey designs [3,51,52,53,54,55,56,57,58,59,60,61,62,63,64,65,66,68,69,70], while one study included an institution-based survey [67]. Sample sizes ranged from 145 teachers in Niger [55] to 845 teachers in Ethiopia [62], and the number of schools included varied widely, with some studies not reporting exact numbers.

Teachers were predominantly female in most studies, with proportions ranging from 52.7% to 100%, and mean ages ranged from 26 to over 46 years, where reported. Study settings typically included primary and secondary schools with both public and private schools represented in Nepal and Saudi Arabia [69,70], with some studies also including nursery schools [66], high schools only [68], or specialized schools for children with intellectual disabilities [56]. Several studies focused exclusively on female teachers [58] or included all educational levels [60].

All studies used self-administered questionnaires to assess knowledge and attitudes toward epilepsy, with many instruments validated or adapted from previous research including a validated Arabic questionnaire consisting of six knowledge items [70] and a multiple-choice structured questionnaire in Nepal [69]. Examples include a 39-item structured Persian questionnaire validated for Iranian teachers [54], the Modified Scale of Attitudes Toward Persons with Epilepsy in Kuwait [57], and structured self-administered questionnaires with reliability indices reported, such as α = 0.83 [52] and α = 0.74 [67]. Other studies used author-developed or adapted tools assessing knowledge, attitudes, and first-aid practices.

Knowledge outcomes varied across studies, ranging from poor to good. Saudi studies reported mean scores indicating good knowledge in some regions [51], moderate knowledge in others [60], and varying familiarity with seizure first-aid practices [53,58] with 57.9% of teachers demonstrating satisfactory knowledge in a recent Saudi survey [70]. In African studies, teachers demonstrated intermediate to moderate knowledge, with widespread misconceptions regarding epilepsy as contagious or linked to demonic possession [52,55,56]. In Nepal, 47.9% of teachers had poor knowledge, while 52.1% demonstrated good knowledge of epilepsy [69]. European studies showed moderate awareness and attitudes, with Italian and Greek teachers reporting reasonable knowledge of causes and treatment but limited understanding of prevalence and curability [3,59]. Knowledge of seizure management was generally limited, with only a fraction of teachers able to provide correct first-aid measures [67,68] (Table 3).

## 4. Discussion

Overall, the findings indicate that teachers’ knowledge and confidence regarding asthma, diabetes, and epilepsy are variable and often suboptimal, with gaps in symptom recognition, management, and first-aid procedures. Female predominance and elementary school settings were common across studies, and self-administered questionnaires were the main assessment method.

When comparing teachers’ knowledge across the three chronic conditions, a consistent pattern emerges in which asthma is generally associated with higher awareness and confidence compared with type 1 diabetes and epilepsy [23]. This may be explained by the higher prevalence of asthma in school-aged children and the more frequent exposure of teachers to asthma-related symptoms and management practices [5]. In contrast, knowledge gaps related to insulin administration, hypoglycemia recognition, and seizure first-aid procedures were more pronounced, particularly for type 1 diabetes and epilepsy, which are less commonly encountered but potentially life-threatening conditions [46,56].

Differences in teachers’ knowledge were also observed across socio-economic settings. Studies conducted in high-income countries more frequently reported higher baseline knowledge levels, greater awareness of school health policies, and better access to school nurses and emergency resources [23,24,42,46]. In middle- and low-income countries, teachers often demonstrated lower confidence and more substantial gaps in disease management, which may reflect limited training opportunities, resource constraints, and reduced access to healthcare support within schools [24,25,44,55]. These findings highlight the influence of health infrastructure and educational investment on school preparedness and underscore the need for context-specific training strategies.

In recent decades, primary and secondary schools have been recognized as key settings for monitoring and managing children with chronic illnesses such as asthma, type 1 diabetes, and epilepsy. Programs like the “asthma-friendly school” in Ontario, Canada, illustrate how schools can provide identification of students with asthma, access to inhalers, emergency protocols, and reduce exposure to triggers [71]. Many teachers emphasized that they have low confidence in managing emergencies and face obstacles such as limited access to medications and poor communication among school staff, including nurses and administrators [72]. Similar strategies can be applied to type 1 diabetes, including glucose monitoring, insulin availability, and teacher education, as well as to epilepsy, with seizure management protocols and trigger avoidance [73].

Teachers often face challenges in managing acute situations and have limited awareness of school policies, highlighting the need for further training and improved communication among staff [73]. This is equally relevant for type 1 diabetes and epilepsy, where well-trained teachers can significantly enhance student safety and quality of life [74]. Importantly, asthma also requires a high level of teacher awareness and preparedness, as severe exacerbations can rapidly become life-threatening if not promptly recognized and managed in the school setting. Schools frequently lack sufficient resources and preparedness, and broader community awareness about these conditions is often inadequate, leading to preventable emergencies and academic disruptions [75].

The presence of school nurses is crucial for improving care for children with chronic conditions. Langton et al. reported that only 24% of teachers were aware of asthma policies compared to 74% of nurses, emphasizing the need for education and communication [1]. Similar benefits are seen for type 1 diabetes and epilepsy, where school nurses provide continuous care and reduce risks of emergencies [76].

Systematic reviews showed that educational interventions in schools reduce symptoms, absences, and hospital visits while improving quality of life for children with asthma [75]. Comparable interventions improve glycemic control and reduce acute episodes in type 1 diabetes, and decrease seizure frequency and enhance safety in epilepsy [77].

The synthesized evidence from studies on asthma, diabetes, and epilepsy underscores a critical need to enhance systemic school preparedness for the management of chronic diseases among students. Strengthening preparedness should be approached through coordinated improvements across key domains, including teacher education and training, health policies and emergency protocols, awareness and inclusion initiatives, intersectoral collaboration and communication, and the establishment of a robust school health infrastructure.

This systematic review is limited by the moderate methodological quality of included studies, as identified through the AXIS assessment. Common issues such as small or unjustified sample sizes, limited questionnaire validation, and incomplete reporting of non-responders may have affected the reliability and generalizability of the findings. Future systematic reviews should consider expanding the scope to include other chronic conditions requiring school-based awareness and emergency preparedness, such as severe food allergies with anaphylaxis risk or rare bleeding disorders, to further inform comprehensive school health policies.

Targeted, evidence-based training should be integrated into both pre-service and in-service teacher education to improve recognition of symptoms, emergency response, and daily management of asthma, diabetes, and epilepsy. Schools should adopt clear health policies that define staff roles, establish emergency procedures, and ensure access to essential medications such as inhalers, insulin, and rescue treatments, supported by regular refresher sessions. Employing trained school nurses or health coordinators is crucial for developing individualized care plans, supervising medication use, and assisting teachers in managing chronic conditions. Strong collaboration between teachers, parents, and healthcare providers is necessary for consistent communication and student support, while school and community awareness programs are essential to reduce stigma and promote inclusion of students with chronic illnesses.

## 5. Conclusions

This systematic review reveals that teachers’ knowledge, preparedness, and confidence in managing asthma, type 1 diabetes, and epilepsy remain insufficient and inconsistent across educational settings. Gaps in symptom recognition, emergency response, and treatment procedures pose risks to student safety and well-being, particularly in schools without dedicated health personnel. Strengthening teacher training, implementing clear health policies, improving communication with families and healthcare professionals, and expanding access to school nursing services are essential steps toward creating safer, more inclusive, and better-prepared learning environments for students with chronic illnesses.

## Figures and Tables

**Table 1:** Characteristics of included studies dealing with the topic of asthma.

Reference Number	Authors and Publication Year	Study Type	Country	Number of Schools	Type of School (Elementary, High School)	Number of Teachers	Gender/Age of Teacher	Type of Questionnaire	Grade of Knowledge About Disease (Percentage)
[23]	YQ Getch et al., 2019	Cross-sectional survey	United States	NA	Elementary and middle schools	593	Gender not reported 20–50 years	Self-administered survey	60.6% reported not feeling well prepared
[24]	Brosso L et al., 2023	Mixed study (sequential explanatory)	Brazil	NA	Elementary school	207	92% female mean age of 40.5	Newcastle Asthma Knowledge Questionnaire	63.8% had unsatisfactory performance
[25]	R M Salih M et al., 2022	Cross-sectional survey	Iraq	NA	Elementary school	103	82.5% female Most were under 40 years (70%)	Self-administered survey, modified from previous authors	72.44% mean total confidence score in managing children with asthma
[26]	Reznik M et al., 2016	Cross-sectional survey	United States	4	Elementary schools	65	95% female Age not stated	Self-administered based on NHLBI guidelines	68% felt uncomfortable assessing/managing asthma attacks
[27]	Nour A et al., 2023	Cross-sectional study	Jordan	20	Elementary schools	200	NA	Self-administered asthma first-aid knowledge questionnaire	Nearly 50% of questions were incorrectly answered
[28]	Adeyeye OO et al., 2018	Cross-sectional survey	Nigeria	54	High school	988	64.1 females Mean age 44.6	Self-administered questionnaire with a knowledge score (maximum 32)	48.1% Poor knowledge
[29]	Mohammadzadeh I et al., 2010	Cross-sectional descriptive analytical study	Iran	80	Elementary school	425	Gender not reported Mean age 42.7	Custom 16-item questionnaire assessing asthma knowledge, max score 16, α = 0.822	Mean knowledge score 12 (intermediate level)
[30]	Ones U et al., 2006	Cross-sectional descriptive study	Turkey	73	Elementary school	792	58.5% female Mean age 38 ± 9 years	questionnaire adapted from KASE-AQ, max score 130	Mean score = 96.7 (around 74.3% of max)—satisfied levels of knowledge
[31]	Awadalla et al., 2024	Analytical cross-sectional	Saudi Arabia	16	Elementary school	384	58.6% female; age mostly 41–50	Self-administered questionnaire, α = 0.793	44.8% unsatisfied knowledge
[32]	Govender et al., 2012	Cross-sectional survey	South Africa	19	Primary schools	226	88.9% female mean age 43 ± 8.2 years	55-item questionnaire on asthma knowledge, administered face-to-face via Mobile Researcher^®^ app	38.5% scored <50% (limited knowledge), 61.5% scored ≥50%
[33]	Canitez et al., 2016	Cross-sectional survey	Turkey	141	Public elementary schools	2779	62.4% female/median age 37.6	Asthma characteristics (unvalidated) and Detailed asthma knowledge, Likert scale (validated)	Mean score for both 60.4%
[34]	Varela et al., 2016	Cross-sectional, observational	Spain	208	Pre-school, primary, secondary	4679	72.6% female, mean age 42.8 ± 10.2 years	Newcastle Asthma Knowledge Questionnaire (NAKQ), validated	Mean score 16/31 (~52% correct); only 6.8% knew main symptoms, 1.5% triggers, 8.6% rescue meds, 32.7% inhaler side effects, 3.8% exercise prevention
[35]	Juliá-Benito et al., 2017	Cross-sectional survey	Spain	NA	Primary, Secondary, High school	2481	Female 69.5% 20–50 years 64.5% 6	25-item self-administered survey developed for study; pilot-tested	97.0% knew what asthma is; 44.5% claimed to know what to do in asthma attack; 54% did not know how to administer asthma medication
[36]	Urrutia-Pereira et al., 2018	Cross-sectional survey	Brazil	NA	Elementary school	177	NA	Portuguese version of Newcastle Asthma Knowledge Questionnaire (NAKQ)	misconceptions on asthma triggers, 4.2% identifying the three main symptoms, 66.1% recognizing bronchial inflammation as the main cause, 91.0% agreeing that children with asthma should not be exposed to smoke, 80.8% knowing preventive medicines must be used daily, and 87.4% acknowledging that severe attacks can lead to hospitalization
[37]	Caruana et al., 2022	Cross-sectional electronic survey	Malta	26	Primary schools	167	NA	Electronic self-reported survey assessing	Mean 5.5 ± 3.3 out of 14 total points (low knowledge)
[38]	Al-Motlaq et al., 2024	Cross-sectional survey	Iraq	NA	Primary school teachers	150	NA	29 multiple true–false questions on asthma facts and management	mean total knowledge score 20.27 ± 2.97, mean confidence score 72.44 ± 13.61
[39]	Sandsund M et al., 2011	cross-sectional survey and qualitative study	Norway	NA	Secondary schools	106	Female 46.2%; mostly 30–39 years 39.6%	Self-administered questionnaire developed from interviews and expert review; Likert-scale items	25.9% reported sufficient knowledge to teach pupils with asthma 89.4% reported need for training
[40]	Alkhamis et al., 2019	Cross-sectional observational survey	Saudi Arabia	NA	Primary/Elementary school	396	Mostly 30–50 years (80.8%), Gender not reported	Self-administered electronic questionnaire	59.6% had high overall asthma awareness; Symptoms: 73.2%; Triggers: 60.9%; Treatment: 69.7%
[41]	Bruzzese et al., 2010	Cross-sectional survey study	United States	25	Public elementary schools	320	NA	Self-reported questionnaire, α = 0.74 to 0.98	Mean total correct score = 68.9%—satisfied levels of knowledge; 93.8% recognized wheezing/shortness of breath as sign, 31.9% correctly identified preventive medication before exercise

**Table 2:** Characteristics of included studies dealing with the topic of diabetes.

Reference Number	Authors and Publication Year	Study Type	Country	Number of Schools	Type of School (Elementary, High School)	Number of Teachers	Gender/Age of Teacher	Type of Questionnaire	Grade of Knowledge About Disease (Percentage)
[42]	Aljefree NM et al., 2023	Cross-sectional study	Saudi Arabia	NA	Elementary and high school	378	Majority female, age 45–54 years	Online survey–self-administered questionnaire (score ranged from to 0–7 for knowledge and 0–5 for attitude)	The average knowledge score was 4.4 (moderate knowledge); Theaverage attitude score was 3.5 (favorable)
[43]	Dixe et al., 2020	Experimental pre-test/post-test study	Portugal	12	Pre-school to high school	129	Not included	self-completed questionnaire, α = 0.926 (score from 0 to 17)	Knowledge scores pre-test was 10.8 (moderate)
[44]	Aycan Z et al., 2012	Cross-sectional study	Turkey	NA	Elementary and high school	1054	73% females, Mean age 38.8 years	Self-administered questionnaire	Moderate knowledge: 47.6% Low knowledge: 32.4%
[45]	Stefanowicz-Bielska et al., 2024	Cross-sectional survey	Poland	NA	Elementary and high school	808	86.4% females, over the 40 years	An original 29-item knowledge test developed by the authors	Median score: 20/29 (69%)—moderate
[46]	Alshammari & Haridi, 2021	Cross-sectional survey	Saudi Arabia	18	Elementary and high school	504	100% female; mean age 39.2	Structured self-administered questionnaire with 20-item Test of Diabetes Knowledge for Teachers	Mean score: 13.2/20 (66.0%)—moderate
[47]	RF Gutzweiler et al., 2020	Cross-sectional survey	Germany	NA	Elementary and high school	678	89% female; age distribution not specified	Structured survey, 5-point Likert scale	Not explicitly stated
[48]	Gutiérrez-Manzanedo et al., 2018	Descriptive observational (cross-sectional)	Spain	44	Public pre-, primary, and secondary schools	765	61.7% female, mean age 44.3 ± 8.8 years	Test of Diabetes Knowledge for Teachers (TDKT	Not enough knowledge: 58% (≤7 points), Basic knowledge: 36.9% (8–12 points), Effective support: 5.1% (13–16 points)
[49]	Gökçe et al., 2019	Cross-sectional survey (online)	Turkey	NA	Public, Private, Specialized	42,349	Mean age 37.9 (20–73), 56% female	55-item online survey (39 knowledge + 16 attitude questions, True/False/Don’t know), α = 0.71	Mean knowledge score 17.7/39 (min −13, max 39); 75% of those aware of DPS reported increase in knowledge
[50]	Bradbury et al., 1983	Cross-sectional survey	United Kingdom	40	Primary and Secondary	97	NA	Self-administered questionnaire (18 knowledge questions + sections on sources & attitudes)	Only 24 teachers (25%) had “adequate” knowledge (score ≥14/18)

**Table 3:** Characteristics of included studies dealing with the topic of epilepsy.

Reference Number	Authors and Publication Year	Study Type	Country	Number of Schools	Type of School (Elementary, High School)	Number of Teachers	Gender/Age of Teacher	Type of Questionnaire	Grade of Knowledge About Disease (Percentage)
[52]	Almarwani et al., 2023	Cross-sectional	Saudi Arabia	NA	Primary, middle, and high schools	394	64.7% female; age older than 40	Self-administered electronic questionnaire, adapted from previous studies	The mean score 28.8—good knowledge
[53]	Elhassan MA et al., 2017	Cross-sectional descriptive study	Sudan	NA	Secondary schools	317	52.7% male; age range 46–60 years	Self-administered questionnaire Knowledge scores scaled 0–14, α = 0.83	mean knowledge score 8.1—intermediate level
[54]	Abulhamail AS et al., 2014	Cross-sectional survey	Saudi Arabia	NA	Primary schools	615	53% female; mean age 36 years	Structured 37-item questionnaire, Likert-scale items included	Only 17% of teachers felt very well informed about epilepsy
[51]	Karimi N et al., 2015	Cross-sectional study	Iran	25	Primary and secondary schools	305	71.5% female, mean age 38.4 years	39-item structured questionnaire, 5-point Likert scale; validated for Persian	82% knew symptoms, 61.3% observed an epileptic fit, 40% reported correct first-aid knowledge
[55]	Assadeck HA et al. 2020	Cross-sectional survey	Niger	NA	Primary and secondary schools	145	64.1% female, mean age: 39.6 years	Self-administered questionnaire	42.1% considered epilepsy a brain disease, 46.2% considered it a contagious disease, 79.3% believed epilepsy is treatable, 11% considered it hereditary, 38.9% viewed it as an impurity, 6.9% thought it was incurable
[56]	Ibinga E et al. 2019	Cross-sectional survey	Gabon	NA	Primary and secondary schools, specialized schools for children with intellectual disabilities	813	57.7% female, mean age 40.1 years	Self-administered questionnaire (19 items)	Brief loss of consciousness: 58.2%, Sudden and brief flexion of neck/limbs: 37.2%, Falling of objects held by child: 33.3%, Contagious: 27.5%, Demonic possession: 16.0%, Brain disease: 43.6%, Neurologic disease: 58.0%
[57]	Al-Hashemi E et al., 2016	Cross-sectional	Kuwait	24	Middle and High Schools	824	55.1% males, mean age 36.9 years	Modified Scale of Attitudes Toward Persons with Epilepsy (ATPE)	knowledge about epilepsy 5 out of 13—weak. attitudes towards epilepsy of 10 out of 15—moderate
[58]	Al-Harbi AF et al., 2018	Cross-sectional survey	Saudi Arabia	NA	Primary female schools	582	100% females, mean age 39	Self-administered, cross-culturally validated questionnaire	79.2% of teachers had heard of epilepsy, 14.3% felt epileptic students should be transferred to special-needs schools, 31.8% expressed ability to give first aid, 27.5% accepted giving prescribed medications to students
[59]	Iannone LF et al., 2021	Cross-sectional survey	Italy	24	Primary and secondary schools	667	91.8% female, mostly older than 35 years	Custom-designed and validated questionnaires in Italian	32.2% obtained their information from personal experience, and 26.5% from formal training or scientific literature in 16.6%. 22.2% were aware of prevalence, 40% of teachers believed epilepsy to be hereditary, 43.9% of teachers believed epilepsy to be incurable.
[60]	Kanjo M et al., 2021	Cross-sectional survey	Saudi Arabia	NA	Primary, intermediate, secondary schools	822	54.1% male, mean age 45	Self-administered validated questionnaire assessing demographic data, knowledge about epilepsy and seizure first aid	69% had moderate knowledge of epilepsy, 16.8% good knowledge, and 14.2% had poor knowledge
[61]	Adal O et al., 2022	Institution-based cross-sectional study	Ethiopia	4	High school	378	Male: 71.7%, Mean age 34	Structured self-administered questionnaire (English version, adapted from previous study, with 10 knowledge and 5 practice questions)	Good knowledge: 41.1% Poor knowledge: 58.9% Good practice: 40.9% Poor practice: 59.1%
[62]	Gebrewold MA et al., 2016	Cross-sectional survey	Ethiopia	20	Primary school	845	58.1 males, mean age 29	Standardized self-administered questionnaire (multi-stage cluster sampling)	Knowledge: 45%—moderate; Attitude: 55%—moderat. Practice: 50%—moderate
[63]	Dantas FG et al., 2001	Cross-sectional study	Brazil	38	Primary, secondary, and tertiary school	300	Female, age not stated	Structured questionnaire with yes/no/don’t know questions	43% of teachers had knowledge of the correct initial procedures during a seizure; other knowledge aspects were lower
[64]	Toudou-Daouda M et al., 2020	Cross-sectional survey	Niger	NA	Primary and secondary school	284	61.6% female, Mean age 37.32 years	Self-administered structured questionnaire in French; 30 questions assessing knowledge (18) and attitudes (12) toward epilepsy	Median score attitudes 60% (18/30)—moderate knowledge attitudes: median 58% (7/12), moderate knowledge
[65]	Babikar HE et al., 2011	Cross-sectional	Sudan	22	Primary and Secondary school	200	56% females, mostly older than 40 years	Pretested, semi-structured, 35-item questionnaire	55% of teachers had knowledge of initial procedures to help a child during a seizure
[66]	Owolabi LF et al., 2014	Cross-sectional survey	Nigeria	NA	Nursery, primary, and secondary schools	200	62% males, median age of 26	Validated 20-item semi-structured self-administered questionnaire	29.5% had fair to good knowledge about epilepsy
[67]	Babore GO et al., 2025	Institutional-based cross-sectional study	Ethiopia	13	Primary and Secondary school	310	61% male, mean age 33.69 years	Structured self-administered questionnaire; reliability α = 0.74	39.4% had good knowledge and 40.2% actually provided at least one appropriate first-aid measure
[68]	Vujisić S et al., 2017	Cross-sectional survey	Montenegro	9	Secondary school	219	74.9% female; mean age 43.38 years	Self-administered 16-item questionnaire	97.7% had heard or read about epilepsy, 57.5% knew someone with epilepsy, 21% had a pupil with epilepsy in their class, 57% had witnessed a seizure, 40% believed epilepsy could be cured, Only 28.3% knew how to provide proper first aid
[3]	Pitta S et al., 2025	Cross-sectional survey study	Greece	NA	Primary schools	546	77.2% females, mean age 40.6 years	Structured questionnaire adapted from an Italian validated study	Aware of epilepsy: 99.3%, Witnessed a seizure: 46.8%, Aware of real prevalence in Greece—10%, Could identify at least one cause: >40%, Knew epilepsy is not psychiatric: >60%, Identified treatment options: >50%, Knew epilepsy is not curable: 33%
[69]	Khanal et al., 2017	Cross-sectional survey	Nepal	6	Primary, secondary; public and private	165	69 M/96 F; age 20–57 (median 29)	Self-administered structured & multiple-choice questionnaire	Poor (<50%): 47.9% Good (≥50%): 52.1%
[70]	Alenazi et al., 2025	Cross-sectional survey	Saudi Arabia	15	Primary, secondary; public and private	366	56% female, 40–49 years 47.3%,	Validated self-administered Arabic questionnaire (6 knowledge questions + demographics)	57.9% had satisfactory knowledge scores (>3/6)

## Data Availability

All data are within this article.

## Supplementary Materials

The following supporting information can be downloaded at: https://www.mdpi.com/article/10.3390/children13010091/s1, Figure S1: AXIS assessment of studies dealing with asthma. Figure S2: AXIS assessment of studies dealing with diabetes. Figure S3: AXIS assessment of studies dealing with epilepsy.
