# Supplementary material for: Examining the Level of Knowledge of Teachers About Asthma, Diabetes and Epilepsy in Children: A Systematic Review
Source: Children (Basel). 2026 Jan 8;13(1):91. doi: 10.3390/children13010091 (PMC12839580; doi:10.3390/children13010091)
Supplement: Supplementary file 1 [file children-13-00091-s001.zip › children-4058600-supplementary.pdf]

AXIS ASSESSMENT

Q1. Were the study aims and objectives explicitly and clearly stated?
Q2. Was the chosen study design suitable for addressing the stated objectives?
Q3. Was the rationale for the selected sample size adequately explained?
Q4. Was the target or reference population clearly specified?
Q5. Was the sampling frame derived from a population that appropriately reflects the target/reference group being studied?
Q6. Did the participant selection process ensure that the sample was representative of the target/reference population?
Q7. Were procedures implemented to identify and classify non-respondents?
Q8. Were the exposure (risk factor) and outcome variables appropriate in relation to the study objectives?
Q9. Were the exposure and outcome variables measured using validated, previously tested, or published instruments or methods?
Q10. Was the basis for determining statistical significance and/or precision measures clearly described?
Q11. Were the study methods, including statistical analyses, described in sufficient detail to allow replication?
Q12. Were the core descriptive data clearly and adequately reported?
Q13. Does the response rate suggest a potential risk of non-response bias?
Q14. Where applicable, was information regarding non-respondents provided?
Q15. Were the study findings internally coherent and consistent?
Q16. Were results reported for all analyses outlined in the methods section?
Q17. Were the interpretations and conclusions drawn by the authors supported by the study findings?
Q18. Did the authors adequately discuss the limitations of the study?
Q19. Were potential funding sources or conflicts of interest disclosed that could influence result interpretation?
Q20. Was appropriate ethical approval obtained and/or informed consent from participants secured?

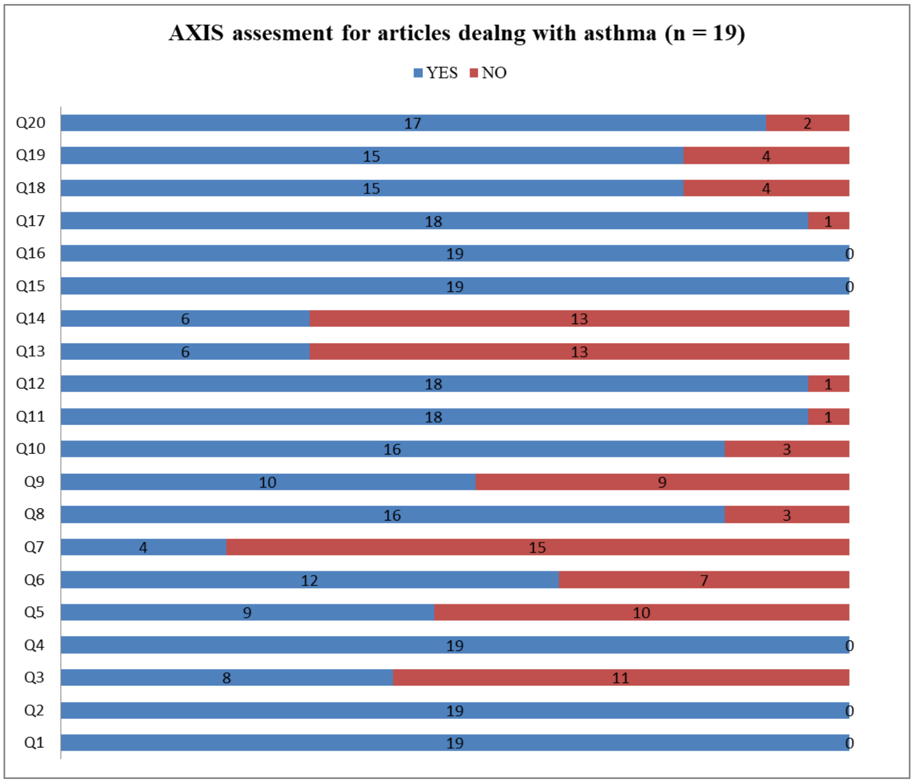

Figure S1: AXIS assessment of studies dealing with asthma

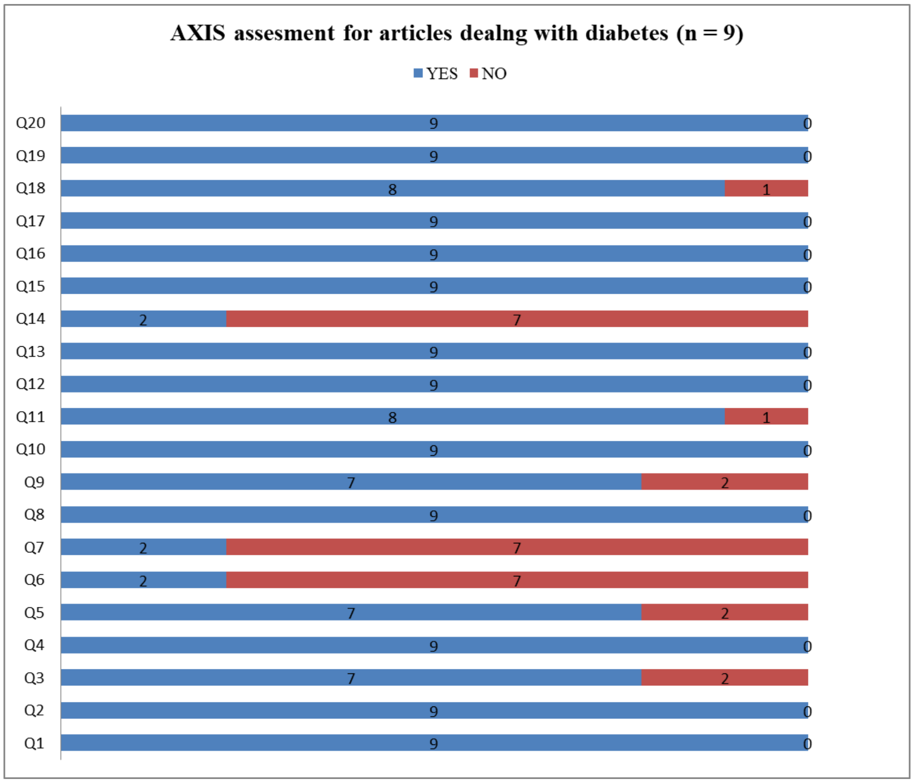

Figure S2: AXIS assessment of studies dealing with diabetes

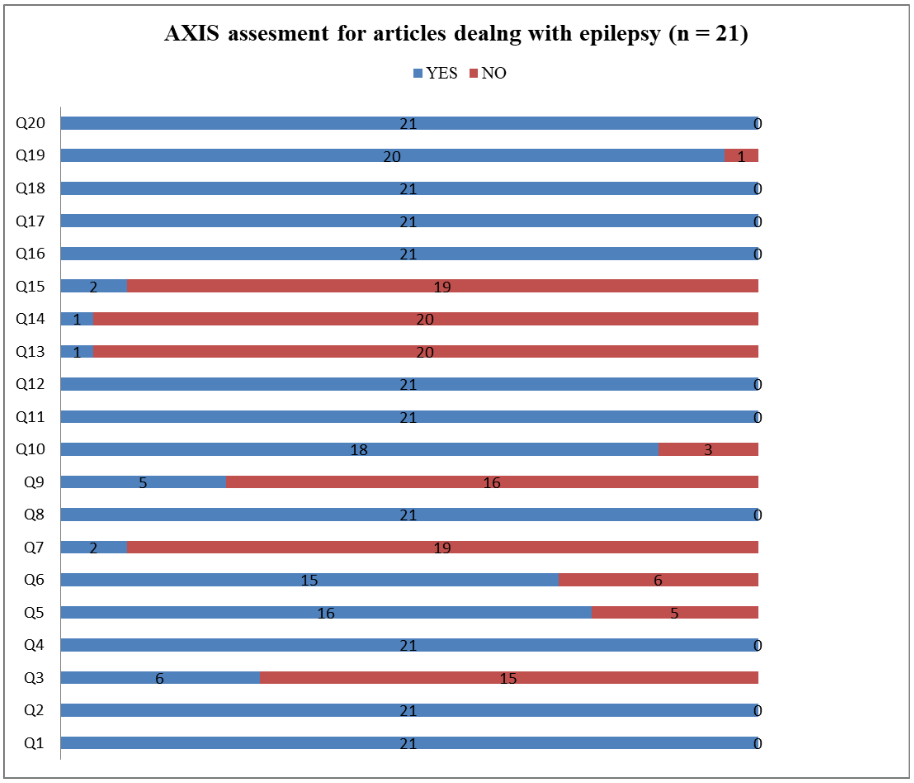

Figure S3: AXIS assessment of studies dealing with epilepsy
